# Crystal structure of (*Z*)-ethyl 2-{5-[(2-benzyl­idene-3-oxo-2,3-di­hydro­benzo[*b*][1,4]thia­zin-4-yl)meth­yl]-1*H*-1,2,3-triazol-1-yl}acetate

**DOI:** 10.1107/S2056989015022987

**Published:** 2015-12-06

**Authors:** M. Ellouz, N. K. Sebbar, E. M. Essassi, Y. Ouzidan, J. T. Mague

**Affiliations:** a Laboratoire de Chimie Organique Hétérocyclique URAC 21, Pôle de Compétence Pharmacochimie, Av. Ibn Battouta, BP 1014, Faculté des Sciences, Université Mohammed V, Rabat, Morocco; b Laboratoire de Chimie Organique Appliquée, Université Sidi Mohamed Ben Abdallah, Faculté des Sciences et Techniques, Route d’immouzzer, BP 2202, Fez, Morocco; c Department of Chemistry, Tulane University, New Orleans, LA 70118, USA

**Keywords:** crystal structure, benzo­thia­zine, triazole, conformation

## Abstract

The title compound, C_22_H_20_N_4_O_3_S, features two fused six-membered rings linked to a 1,2,3-triazole ring which is attached to an ethyl acetate group. The heterocycle in the benzo­thia­zine residue has an envelope conformation with the S atom being the flap. The conformation of the ethyl acetate side chain, which is directed to the same side of the mol­ecule as the C_6_ ring of the fused-ring system, may be partially established by a pair of weak intra­molecular C—H⋯O(carbon­yl) inter­actions. The three-dimensional packing is aided by inter­molecular C—H⋯O and C—H⋯N inter­actions.

## Related literature   

For the biological activity of 1,4-benzo­thia­zine derivatives, see: Goyal *et al.* (2013 ▸); Gupta *et al.* (2011 ▸); Gautam *et al.* (2013 ▸); Deshmukh & Mulik (2004 ▸); Kumar *et al.* (2010 ▸); Hans *et al.* (2008 ▸); Gao *et al.* (2005 ▸); Bakavoli *et al.* (2007 ▸). For applications of 1,4-benzo­thia­zine derivatives, see: Podsiadły *et al.* (2009 ▸); Hong *et al.* (2008 ▸). For structures of 1,4-benzo­thia­zine derivatives, see: Sebbar *et al.* (2014 ▸).
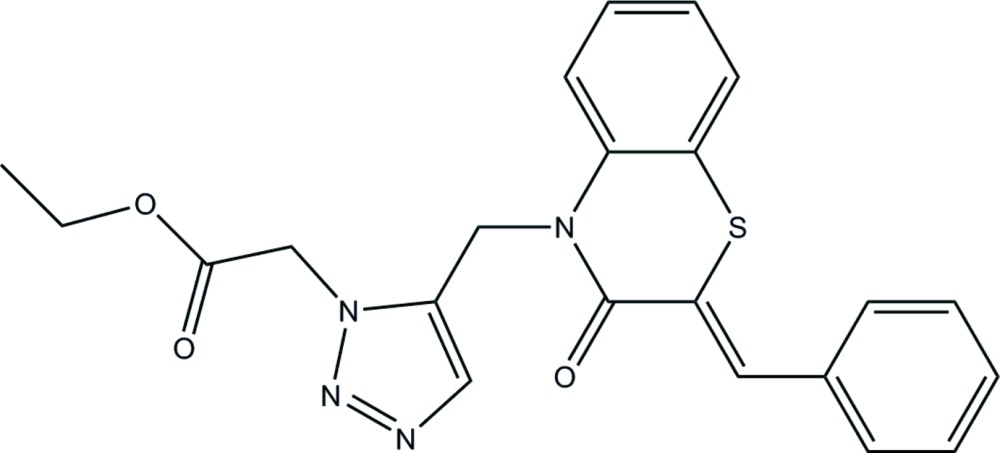


## Experimental   

### Crystal data  

C_22_H_20_N_4_O_3_S
*M* *_r_* = 420.48
Monoclinic, 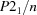

*a* = 9.9767 (6) Å
*b* = 8.7342 (5) Å
*c* = 23.1027 (14) Å
β = 94.508 (1)°
*V* = 2006.9 (2) Å^3^
*Z* = 4
Mo *K*α radiation
μ = 0.19 mm^−1^
*T* = 150 K
0.32 × 0.28 × 0.25 mm

### Data collection  

Bruker SMART APEX CCD diffractometer
Absorption correction: multi-scan (*SADABS*; Bruker, 2015 ▸) *T*_min_ = 0.86, *T*_max_ = 0.95
37542 measured reflections
5341 independent reflections
4380 reflections with *I* > 2σ(*I*)
*R*_int_ = 0.039

### Refinement  

*R*[*F* ^2^ > 2σ(*F* ^2^)] = 0.043
*wR*(*F* ^2^) = 0.117
*S* = 1.04
5341 reflections
272 parameters
H-atom parameters constrained
Δρ_max_ = 0.42 e Å^−3^
Δρ_min_ = −0.26 e Å^−3^

### 

Data collection: *APEX2* (Bruker, 2015 ▸); cell refinement: *SAINT* (Bruker, 2015 ▸); data reduction: *SAINT*; program(s) used to solve structure: *SHELXT* (Sheldrick, 2015*a* ▸); program(s) used to refine structure: *SHELXL* (Sheldrick, 2015*b* ▸); molecular graphics: *DIAMOND* (Brandenburg & Putz, 2012 ▸); software used to prepare material for publication: *SHELXTL* (Sheldrick, 2008 ▸).

## Supplementary Material

Crystal structure: contains datablock(s) global, I. DOI: 10.1107/S2056989015022987/tk5410sup1.cif

Structure factors: contains datablock(s) I. DOI: 10.1107/S2056989015022987/tk5410Isup2.hkl

Supporting information file. DOI: 10.1107/S2056989015022987/tk5410Isup3.cml

**Fig. 1.**
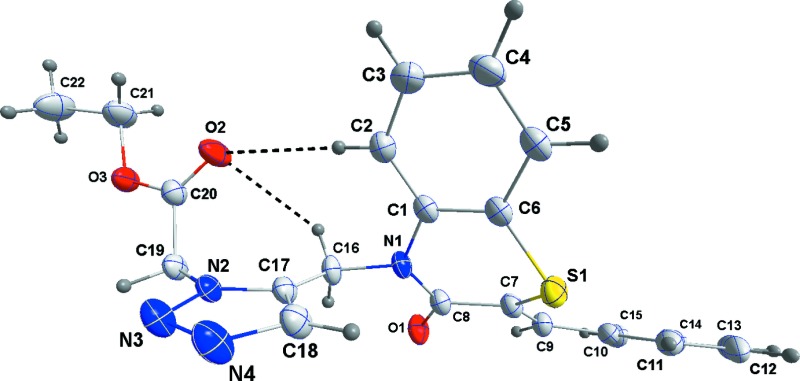
The title molecule showing the labeling scheme and 50% probability ellipsoids. Intramolecular C—H···O interactions are shown by dotted lines.

**Fig. 2.**
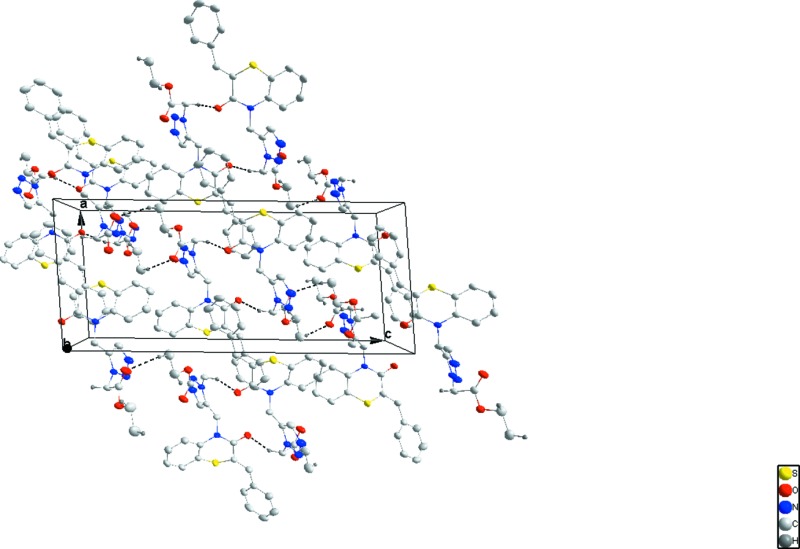
Packing viewed down the b axis. Intermolecular C—H···O interactions are shown by dotted lines.

. DOI: 10.1107/S2056989015022987/tk5410fig1.tif
The title mol­ecule showing the labeling scheme and 50% probability ellipsoids. Intra­molecular C—H⋯O inter­actions are shown by dotted lines.

b . DOI: 10.1107/S2056989015022987/tk5410fig2.tif
Packing viewed down the *b* axis. Inter­molecular C—H⋯O inter­actions are shown by dotted lines.

CCDC reference: 1439697

Additional supporting information:  crystallographic information; 3D view; checkCIF report

## Figures and Tables

**Table 1:** Hydrogen-bond geometry (Å, °)

*D*—H⋯*A*	*D*—H	H⋯*A*	*D*⋯*A*	*D*—H⋯*A*
C2—H2⋯O2	0.95	2.55	3.4726 (18)	163
C16—H16*B*⋯O2	0.99	2.58	3.2981 (19)	129
C19—H19*B*⋯O1^i^	0.99	2.39	3.2547 (18)	146
C21—H21*A*⋯N4^ii^	0.99	2.57	3.526 (2)	162
C22—H22*B*⋯O2^iii^	0.98	2.59	3.521 (2)	159

Symmetry codes: (i) 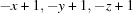; (ii) 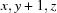; (iii) 
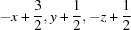
.

## supplementary crystallographic information

### S1. Comment

4*H*-1,4-Benzothiazines possess a wide spectrum of biological and pharmacological activities due to the presence of a fold along the nitrogen···sulfur axis which is considered to be one of the structural features responsible for their activities (Gupta *et al.*, 2011). During the past two decades, we have found a growing interest in 1,4-benzothiazines. In fact, the 1,4-benzothiazines are the best known to possess biologically diverse activities (Goyal *et al.*, 2013) such as antimicrobial, (Gautam *et al.*, 2013) antifungal (Hans *et al.*, 2008), antioxidant agents (Kumar *et al.*, 2010), inhibitors of beta-ribosidases (Gao *et al.*, 2005), potential vasodilators (Deshmukh *et al.*, 2004) and as potent lipoxygenase inhibitors (Bakavoli *et al.*, 2007). 1,4- Benzothiazines are the basis for novel dyes (Podsiadły *et al.*, 2009) and behave as semiconductors (Hong *et al.*, 2008).

As a continuation of our research devoted to the development of substituted 1,4-benzothiazine derivatives (Sebbar *et al.*, 2014), we report the synthesis of a new 1,4-benzothiazine derivative which is built from two fused six-membered rings linked to a 1,2,3-triazole ring which is attached to an ethylacetate group.

The conformation of the side chain may be partially established by the weak, intramolecular C16—H16B···O2 and C2—H2···O2 interactions (Fig. 1 and Table 1). The six-membered heterocyclic ring has puckering parameters Q = 0.5154 (11) Å, θ = 108.31 (14)° and φ = 162.81 (17)°. The pendant phenyl ring (C10–C15) makes a dihedral angle of 53.26 (5)° with the ring C1–C6 while the dihedral angle between the ring C1–C6 and the triazolyl ring is 76.31 (5)°. The packing is aided by intermolecular C—H···O and C—H···N interactions (Figs 2 and Table 1).

### S2. Experimental

To a solution of 2-benzylidene-4-(prop-2-yn-1-yl)-2*H*-1,4-benzothiazin-3-one (0.2 g, 0.68 mmol) in ethanol (15 ml) was added ethyl azido-acetate (0.13 g, 1.03 mmol). The mixture was stirred under reflux for 24 h. After completion of the reaction (monitored by TLC), the solution was concentrated and the residue was purified by column chromatography on silica gel by using a mixture (hexane/ethyl acetate 9/1). Crystals were obtained when the solvent was allowed to evaporate. The solid product was purified by recrystallization from ethanol to afford yellow crystals in 14% yield.

### S3. Refinement

Carbon-bound H-atoms were placed in calculated positions (C—H = 0.95 to 0.99 Å) and were included in the refinement in the riding model approximation with *U*_iso_(H) = 1.2–1.5*U*_eq_(C).

### Crystal data

**Table:**

C_22_H_20_N_4_O_3_S	*F*(000) = 880
*M**_r_* = 420.48	*D*_x_ = 1.392 Mg m^−^^3^
Monoclinic, *P*2_1_/*n*	Mo *K*α radiation, λ = 0.71073 Å
*a* = 9.9767 (6) Å	Cell parameters from 9992 reflections
*b* = 8.7342 (5) Å	θ = 2.2–29.0°
*c* = 23.1027 (14) Å	µ = 0.19 mm^−^^1^
β = 94.508 (1)°	*T* = 150 K
*V* = 2006.9 (2) Å^3^	Block, colourless
*Z* = 4	0.32 × 0.28 × 0.25 mm

### Data collection

**Table:**

Bruker SMART APEX CCD diffractometer	5341 independent reflections
Radiation source: fine-focus sealed tube	4380 reflections with *I* > 2σ(*I*)
Graphite monochromator	*R*_int_ = 0.039
Detector resolution: 8.3333 pixels mm^-1^	θ_max_ = 29.1°, θ_min_ = 1.8°
φ and ω scans	*h* = −13→13
Absorption correction: multi-scan (*SADABS*; Bruker, 2015)	*k* = −11→11
*T*_min_ = 0.86, *T*_max_ = 0.95	*l* = −30→31
37542 measured reflections	

### Refinement

**Table:**

Refinement on *F*^2^	Primary atom site location: structure-invariant direct methods
Least-squares matrix: full	Secondary atom site location: difference Fourier map
*R*[*F*^2^ > 2σ(*F*^2^)] = 0.043	Hydrogen site location: inferred from neighbouring sites
*wR*(*F*^2^) = 0.117	H-atom parameters constrained
*S* = 1.04	*w* = 1/[σ^2^(*F*_o_^2^) + (0.0606*P*)^2^ + 0.6062*P*] where *P* = (*F*_o_^2^ + 2*F*_c_^2^)/3
5341 reflections	(Δ/σ)_max_ < 0.001
272 parameters	Δρ_max_ = 0.42 e Å^−^^3^
0 restraints	Δρ_min_ = −0.26 e Å^−^^3^

### Special details

**Table:**

Experimental. The diffraction data were obtained from 3 sets of 400 frames, each of width 0.5° in ω, colllected at φ = 0.00, 90.00 and 180.00° and 2 sets of 800 frames, each of width 0.45° in φ, collected at ω = −30.00 and 210.00°. The scan time was 8 sec/frame.
Geometry. All e.s.d.'s (except the e.s.d. in the dihedral angle between two l.s. planes) are estimated using the full covariance matrix. The cell e.s.d.'s are taken into account individually in the estimation of e.s.d.'s in distances, angles and torsion angles; correlations between e.s.d.'s in cell parameters are only used when they are defined by crystal symmetry. An approximate (isotropic) treatment of cell e.s.d.'s is used for estimating e.s.d.'s involving l.s. planes.
Refinement. Refinement of *F*^2^ against ALL reflections. The weighted *R*-factor *wR* and goodness of fit *S* are based on *F*^2^, conventional *R*-factors *R* are based on *F*, with *F* set to zero for negative *F*^2^. The threshold expression of *F*^2^ > σ(*F*^2^) is used only for calculating *R*-factors(gt) *etc*. and is not relevant to the choice of reflections for refinement. *R*-factors based on *F*^2^ are statistically about twice as large as those based on *F*, and *R*- factors based on ALL data will be even larger. H-atoms attached to carbon were placed in calculated positions (C—H = 0.95 − 0.99 Å). All were included as riding contributions with isotropic displacement parameters 1.2 − 1.5 times those of the attached atoms.

### Fractional atomic coordinates and isotropic or equivalent isotropic displacement parameters (Å^2^)

**Table:**

	*x*	*y*	*z*	*U*_iso_*/*U*_eq_	
S1	0.06354 (3)	0.15135 (4)	0.40087 (2)	0.02889 (10)	
O1	0.31909 (10)	0.34881 (14)	0.51171 (4)	0.0355 (3)	
O2	0.61888 (11)	0.57371 (14)	0.32065 (5)	0.0404 (3)	
O3	0.83615 (10)	0.63206 (12)	0.34309 (5)	0.0307 (2)	
N1	0.31595 (11)	0.34444 (14)	0.41352 (5)	0.0262 (2)	
N2	0.66451 (10)	0.28821 (13)	0.37357 (5)	0.0243 (2)	
N3	0.70889 (12)	0.16441 (15)	0.34542 (6)	0.0326 (3)	
N4	0.60856 (13)	0.06776 (15)	0.33911 (6)	0.0374 (3)	
C1	0.24452 (13)	0.34916 (16)	0.35742 (6)	0.0254 (3)	
C2	0.29275 (14)	0.43188 (18)	0.31212 (7)	0.0322 (3)	
H2	0.3737	0.4888	0.3184	0.039*	
C3	0.22313 (15)	0.4319 (2)	0.25761 (7)	0.0364 (3)	
H3	0.2568	0.4895	0.2270	0.044*	
C4	0.10540 (15)	0.34906 (19)	0.24722 (7)	0.0350 (3)	
H4	0.0590	0.3489	0.2097	0.042*	
C5	0.05591 (14)	0.26665 (17)	0.29200 (6)	0.0296 (3)	
H5	−0.0256	0.2108	0.2854	0.036*	
C6	0.12545 (13)	0.26549 (16)	0.34678 (6)	0.0250 (3)	
C7	0.11667 (13)	0.26130 (16)	0.46180 (6)	0.0256 (3)	
C8	0.25729 (13)	0.32240 (17)	0.46472 (6)	0.0265 (3)	
C9	0.04663 (13)	0.27766 (16)	0.50890 (6)	0.0275 (3)	
H9	0.0936	0.3301	0.5403	0.033*	
C10	−0.08942 (13)	0.22868 (16)	0.52021 (6)	0.0274 (3)	
C11	−0.16582 (15)	0.11893 (19)	0.48788 (7)	0.0333 (3)	
H11	−0.1284	0.0672	0.4568	0.040*	
C12	−0.29624 (16)	0.0855 (2)	0.50125 (8)	0.0413 (4)	
H12	−0.3479	0.0126	0.4786	0.050*	
C13	−0.35135 (16)	0.1574 (2)	0.54703 (9)	0.0442 (4)	
H13	−0.4413	0.1359	0.5552	0.053*	
C14	−0.27500 (16)	0.2608 (2)	0.58095 (8)	0.0422 (4)	
H14	−0.3113	0.3075	0.6134	0.051*	
C15	−0.14533 (15)	0.29617 (18)	0.56757 (7)	0.0342 (3)	
H15	−0.0938	0.3675	0.5910	0.041*	
C16	0.45934 (12)	0.38457 (18)	0.41794 (6)	0.0279 (3)	
H16A	0.4944	0.3859	0.4592	0.033*	
H16B	0.4714	0.4879	0.4016	0.033*	
C17	0.53491 (12)	0.27008 (16)	0.38550 (6)	0.0247 (3)	
C18	0.50109 (14)	0.12925 (17)	0.36318 (7)	0.0309 (3)	
H18	0.4157	0.0818	0.3643	0.037*	
C19	0.75516 (12)	0.41430 (16)	0.38687 (6)	0.0263 (3)	
H19A	0.8488	0.3793	0.3842	0.032*	
H19B	0.7462	0.4486	0.4272	0.032*	
C20	0.72677 (13)	0.54792 (17)	0.34581 (6)	0.0271 (3)	
C21	0.82319 (18)	0.7661 (2)	0.30459 (8)	0.0434 (4)	
H21A	0.7736	0.8489	0.3229	0.052*	
H21B	0.7735	0.7384	0.2673	0.052*	
C22	0.96129 (19)	0.8177 (2)	0.29460 (9)	0.0494 (5)	
H22A	1.0087	0.8471	0.3317	0.074*	
H22B	0.9564	0.9059	0.2683	0.074*	
H22C	1.0100	0.7341	0.2772	0.074*	

### Atomic displacement parameters (Å^2^)

**Table:**

	*U*^11^	*U*^22^	*U*^33^	*U*^12^	*U*^13^	*U*^23^
S1	0.02741 (17)	0.03138 (19)	0.02719 (18)	−0.00666 (13)	−0.00227 (13)	−0.00326 (13)
O1	0.0253 (5)	0.0528 (7)	0.0275 (5)	−0.0067 (4)	−0.0030 (4)	−0.0082 (5)
O2	0.0279 (5)	0.0415 (6)	0.0497 (7)	−0.0022 (5)	−0.0106 (5)	0.0113 (5)
O3	0.0245 (5)	0.0307 (5)	0.0366 (6)	−0.0035 (4)	−0.0004 (4)	0.0053 (4)
N1	0.0167 (5)	0.0345 (6)	0.0269 (6)	−0.0013 (4)	−0.0020 (4)	−0.0043 (5)
N2	0.0179 (5)	0.0254 (6)	0.0289 (6)	0.0010 (4)	−0.0023 (4)	−0.0022 (4)
N3	0.0252 (6)	0.0314 (7)	0.0405 (7)	0.0047 (5)	−0.0021 (5)	−0.0087 (5)
N4	0.0284 (6)	0.0304 (7)	0.0517 (8)	0.0027 (5)	−0.0068 (5)	−0.0102 (6)
C1	0.0198 (6)	0.0292 (7)	0.0266 (7)	0.0027 (5)	−0.0019 (5)	−0.0027 (5)
C2	0.0248 (6)	0.0369 (8)	0.0344 (8)	−0.0022 (6)	−0.0003 (5)	0.0014 (6)
C3	0.0326 (7)	0.0433 (9)	0.0329 (8)	0.0008 (6)	0.0004 (6)	0.0087 (7)
C4	0.0315 (7)	0.0422 (9)	0.0297 (7)	0.0049 (6)	−0.0073 (6)	0.0038 (6)
C5	0.0222 (6)	0.0347 (8)	0.0306 (7)	0.0019 (5)	−0.0061 (5)	−0.0015 (6)
C6	0.0207 (6)	0.0273 (7)	0.0265 (6)	0.0024 (5)	−0.0012 (5)	−0.0017 (5)
C7	0.0213 (6)	0.0274 (7)	0.0274 (7)	−0.0009 (5)	−0.0032 (5)	−0.0022 (5)
C8	0.0203 (6)	0.0303 (7)	0.0284 (7)	−0.0002 (5)	−0.0008 (5)	−0.0044 (5)
C9	0.0230 (6)	0.0291 (7)	0.0297 (7)	−0.0036 (5)	−0.0018 (5)	−0.0012 (5)
C10	0.0221 (6)	0.0297 (7)	0.0299 (7)	−0.0011 (5)	−0.0016 (5)	0.0068 (6)
C11	0.0312 (7)	0.0376 (8)	0.0303 (7)	−0.0079 (6)	−0.0035 (6)	0.0063 (6)
C12	0.0308 (7)	0.0465 (9)	0.0444 (9)	−0.0142 (7)	−0.0114 (7)	0.0170 (8)
C13	0.0229 (7)	0.0488 (10)	0.0605 (11)	−0.0008 (6)	0.0014 (7)	0.0215 (9)
C14	0.0324 (8)	0.0389 (9)	0.0570 (11)	0.0046 (7)	0.0144 (7)	0.0082 (8)
C15	0.0299 (7)	0.0309 (8)	0.0421 (8)	0.0006 (6)	0.0055 (6)	0.0022 (6)
C16	0.0170 (6)	0.0350 (7)	0.0312 (7)	−0.0032 (5)	−0.0006 (5)	−0.0067 (6)
C17	0.0182 (6)	0.0285 (7)	0.0266 (6)	−0.0005 (5)	−0.0032 (5)	0.0002 (5)
C18	0.0238 (6)	0.0287 (7)	0.0389 (8)	−0.0019 (5)	−0.0052 (6)	−0.0025 (6)
C19	0.0175 (5)	0.0292 (7)	0.0315 (7)	−0.0022 (5)	−0.0033 (5)	0.0003 (5)
C20	0.0238 (6)	0.0290 (7)	0.0280 (7)	−0.0003 (5)	−0.0004 (5)	−0.0022 (5)
C21	0.0411 (9)	0.0374 (9)	0.0515 (10)	−0.0001 (7)	0.0013 (7)	0.0147 (8)
C22	0.0488 (10)	0.0489 (10)	0.0496 (10)	−0.0152 (8)	−0.0022 (8)	0.0153 (9)

### Geometric parameters (Å, º)

**Table:**

S1—C6	1.7488 (15)	C9—H9	0.9500
S1—C7	1.7513 (14)	C10—C15	1.397 (2)
O1—C8	1.2275 (16)	C10—C11	1.403 (2)
O2—C20	1.2037 (16)	C11—C12	1.392 (2)
O3—C20	1.3212 (16)	C11—H11	0.9500
O3—C21	1.4696 (19)	C12—C13	1.380 (3)
N1—C8	1.3738 (18)	C12—H12	0.9500
N1—C1	1.4297 (17)	C13—C14	1.384 (3)
N1—C16	1.4686 (16)	C13—H13	0.9500
N2—C17	1.3523 (16)	C14—C15	1.388 (2)
N2—N3	1.3540 (17)	C14—H14	0.9500
N2—C19	1.4427 (17)	C15—H15	0.9500
N3—N4	1.3088 (18)	C16—C17	1.4897 (19)
N4—C18	1.357 (2)	C16—H16A	0.9900
C1—C2	1.389 (2)	C16—H16B	0.9900
C1—C6	1.3999 (18)	C17—C18	1.366 (2)
C2—C3	1.390 (2)	C18—H18	0.9500
C2—H2	0.9500	C19—C20	1.516 (2)
C3—C4	1.384 (2)	C19—H19A	0.9900
C3—H3	0.9500	C19—H19B	0.9900
C4—C5	1.383 (2)	C21—C22	1.485 (2)
C4—H4	0.9500	C21—H21A	0.9900
C5—C6	1.3946 (18)	C21—H21B	0.9900
C5—H5	0.9500	C22—H22A	0.9800
C7—C9	1.346 (2)	C22—H22B	0.9800
C7—C8	1.4975 (18)	C22—H22C	0.9800
C9—C10	1.4660 (19)		
			
C6—S1—C7	99.22 (7)	C13—C12—H12	119.7
C20—O3—C21	115.99 (11)	C11—C12—H12	119.7
C8—N1—C1	124.67 (11)	C12—C13—C14	119.71 (15)
C8—N1—C16	116.88 (11)	C12—C13—H13	120.1
C1—N1—C16	118.01 (11)	C14—C13—H13	120.1
C17—N2—N3	111.00 (11)	C13—C14—C15	120.01 (17)
C17—N2—C19	129.74 (12)	C13—C14—H14	120.0
N3—N2—C19	119.26 (11)	C15—C14—H14	120.0
N4—N3—N2	107.01 (11)	C14—C15—C10	121.12 (16)
N3—N4—C18	108.73 (12)	C14—C15—H15	119.4
C2—C1—C6	118.60 (13)	C10—C15—H15	119.4
C2—C1—N1	121.38 (12)	N1—C16—C17	109.49 (11)
C6—C1—N1	119.99 (12)	N1—C16—H16A	109.8
C1—C2—C3	120.32 (14)	C17—C16—H16A	109.8
C1—C2—H2	119.8	N1—C16—H16B	109.8
C3—C2—H2	119.8	C17—C16—H16B	109.8
C4—C3—C2	120.94 (15)	H16A—C16—H16B	108.2
C4—C3—H3	119.5	N2—C17—C18	103.91 (12)
C2—C3—H3	119.5	N2—C17—C16	123.63 (12)
C5—C4—C3	119.36 (14)	C18—C17—C16	132.43 (12)
C5—C4—H4	120.3	N4—C18—C17	109.36 (13)
C3—C4—H4	120.3	N4—C18—H18	125.3
C4—C5—C6	120.08 (13)	C17—C18—H18	125.3
C4—C5—H5	120.0	N2—C19—C20	111.79 (11)
C6—C5—H5	120.0	N2—C19—H19A	109.3
C5—C6—C1	120.69 (13)	C20—C19—H19A	109.3
C5—C6—S1	118.30 (11)	N2—C19—H19B	109.3
C1—C6—S1	120.97 (10)	C20—C19—H19B	109.3
C9—C7—C8	118.01 (12)	H19A—C19—H19B	107.9
C9—C7—S1	124.36 (10)	O2—C20—O3	125.70 (14)
C8—C7—S1	117.06 (10)	O2—C20—C19	124.20 (13)
O1—C8—N1	121.12 (12)	O3—C20—C19	110.09 (11)
O1—C8—C7	120.70 (13)	O3—C21—C22	107.30 (14)
N1—C8—C7	118.17 (11)	O3—C21—H21A	110.3
C7—C9—C10	131.28 (13)	C22—C21—H21A	110.3
C7—C9—H9	114.4	O3—C21—H21B	110.3
C10—C9—H9	114.4	C22—C21—H21B	110.3
C15—C10—C11	118.13 (13)	H21A—C21—H21B	108.5
C15—C10—C9	116.66 (13)	C21—C22—H22A	109.5
C11—C10—C9	125.20 (14)	C21—C22—H22B	109.5
C12—C11—C10	120.22 (16)	H22A—C22—H22B	109.5
C12—C11—H11	119.9	C21—C22—H22C	109.5
C10—C11—H11	119.9	H22A—C22—H22C	109.5
C13—C12—C11	120.69 (16)	H22B—C22—H22C	109.5
			
C17—N2—N3—N4	0.23 (16)	C8—C7—C9—C10	−178.60 (14)
C19—N2—N3—N4	179.65 (12)	S1—C7—C9—C10	−7.6 (2)
N2—N3—N4—C18	−0.15 (17)	C7—C9—C10—C15	−164.25 (15)
C8—N1—C1—C2	152.35 (14)	C7—C9—C10—C11	16.7 (3)
C16—N1—C1—C2	−19.71 (19)	C15—C10—C11—C12	3.4 (2)
C8—N1—C1—C6	−29.6 (2)	C9—C10—C11—C12	−177.55 (14)
C16—N1—C1—C6	158.31 (13)	C10—C11—C12—C13	−1.3 (2)
C6—C1—C2—C3	0.5 (2)	C11—C12—C13—C14	−1.7 (2)
N1—C1—C2—C3	178.50 (14)	C12—C13—C14—C15	2.4 (3)
C1—C2—C3—C4	−0.4 (2)	C13—C14—C15—C10	−0.2 (3)
C2—C3—C4—C5	0.7 (2)	C11—C10—C15—C14	−2.7 (2)
C3—C4—C5—C6	−1.0 (2)	C9—C10—C15—C14	178.17 (14)
C4—C5—C6—C1	1.0 (2)	C8—N1—C16—C17	122.76 (13)
C4—C5—C6—S1	−176.62 (12)	C1—N1—C16—C17	−64.56 (16)
C2—C1—C6—C5	−0.7 (2)	N3—N2—C17—C18	−0.20 (15)
N1—C1—C6—C5	−178.82 (13)	C19—N2—C17—C18	−179.55 (13)
C2—C1—C6—S1	176.83 (11)	N3—N2—C17—C16	177.86 (13)
N1—C1—C6—S1	−1.25 (18)	C19—N2—C17—C16	−1.5 (2)
C7—S1—C6—C5	−149.26 (11)	N1—C16—C17—N2	169.08 (12)
C7—S1—C6—C1	33.12 (13)	N1—C16—C17—C18	−13.5 (2)
C6—S1—C7—C9	144.28 (13)	N3—N4—C18—C17	0.03 (18)
C6—S1—C7—C8	−44.59 (12)	N2—C17—C18—N4	0.11 (16)
C1—N1—C8—O1	−165.70 (14)	C16—C17—C18—N4	−177.71 (15)
C16—N1—C8—O1	6.4 (2)	C17—N2—C19—C20	−77.52 (18)
C1—N1—C8—C7	15.8 (2)	N3—N2—C19—C20	103.18 (14)
C16—N1—C8—C7	−172.03 (12)	C21—O3—C20—O2	−1.6 (2)
C9—C7—C8—O1	18.9 (2)	C21—O3—C20—C19	179.90 (13)
S1—C7—C8—O1	−152.77 (12)	N2—C19—C20—O2	26.0 (2)
C9—C7—C8—N1	−162.59 (13)	N2—C19—C20—O3	−155.45 (12)
S1—C7—C8—N1	25.70 (17)	C20—O3—C21—C22	−164.23 (15)

### Hydrogen-bond geometry (Å, º)

**Table:**

*D*—H···*A*	*D*—H	H···*A*	*D*···*A*	*D*—H···*A*
C2—H2···O2	0.95	2.55	3.4726 (18)	163
C16—H16*B*···O2	0.99	2.58	3.2981 (19)	129
C19—H19*B*···O1^i^	0.99	2.39	3.2547 (18)	146
C21—H21*A*···N4^ii^	0.99	2.57	3.526 (2)	162
C22—H22*B*···O2^iii^	0.98	2.59	3.521 (2)	159

Symmetry codes: (i) −*x*+1, −*y*+1, −*z*+1; (ii) *x*, *y*+1, *z*; (iii) −*x*+3/2, *y*+1/2, −*z*+1/2.
