# Supplementary figures and images for: Crystal structure of (Z)-ethyl 2-{5-[(2-benzyl­idene-3-oxo-2,3-di­hydro­benzo[b][1,4]thia­zin-4-yl)meth­yl]-1H-1,2,3-triazol-1-yl}acetate
Source: Acta Crystallogr E Crystallogr Commun. 2015 Dec 6;71(Pt 12):o1022–3. doi: 10.1107/S2056989015022987 (PMC4719958; doi:10.1107/S2056989015022987)

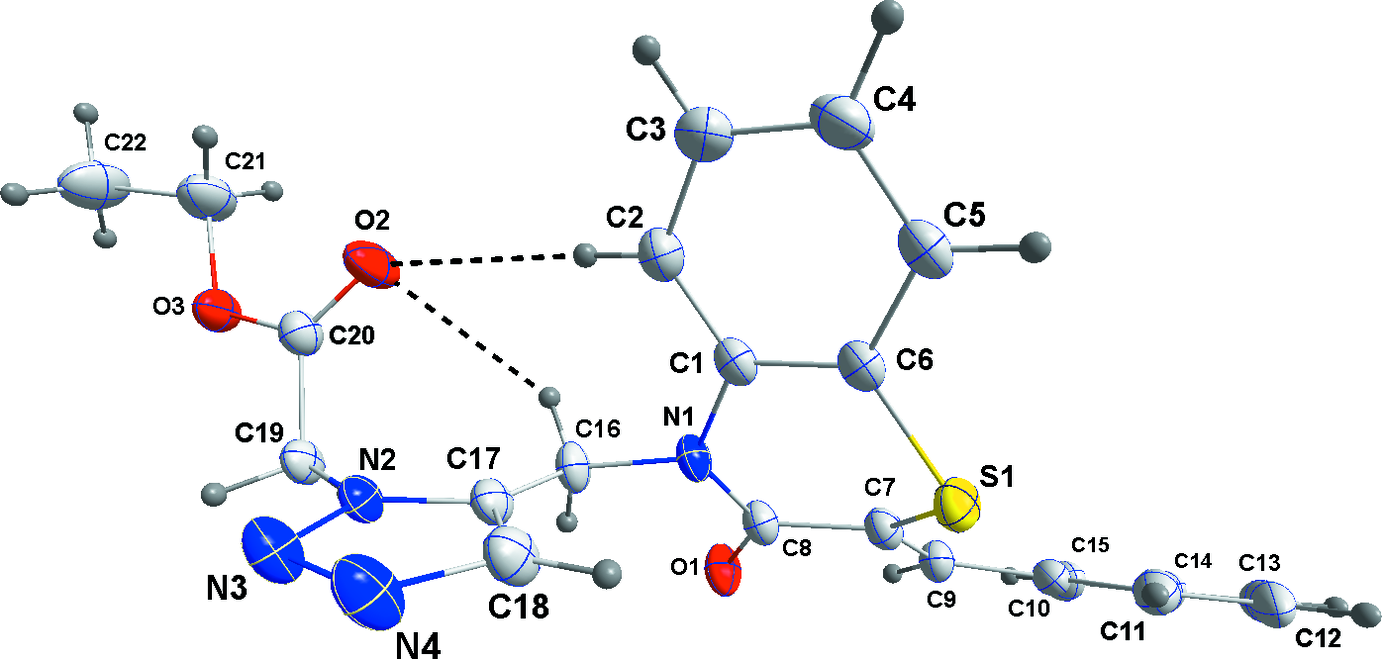

Supplement: Supplementary file 4 [file e-71-o1022-fig1.tif]

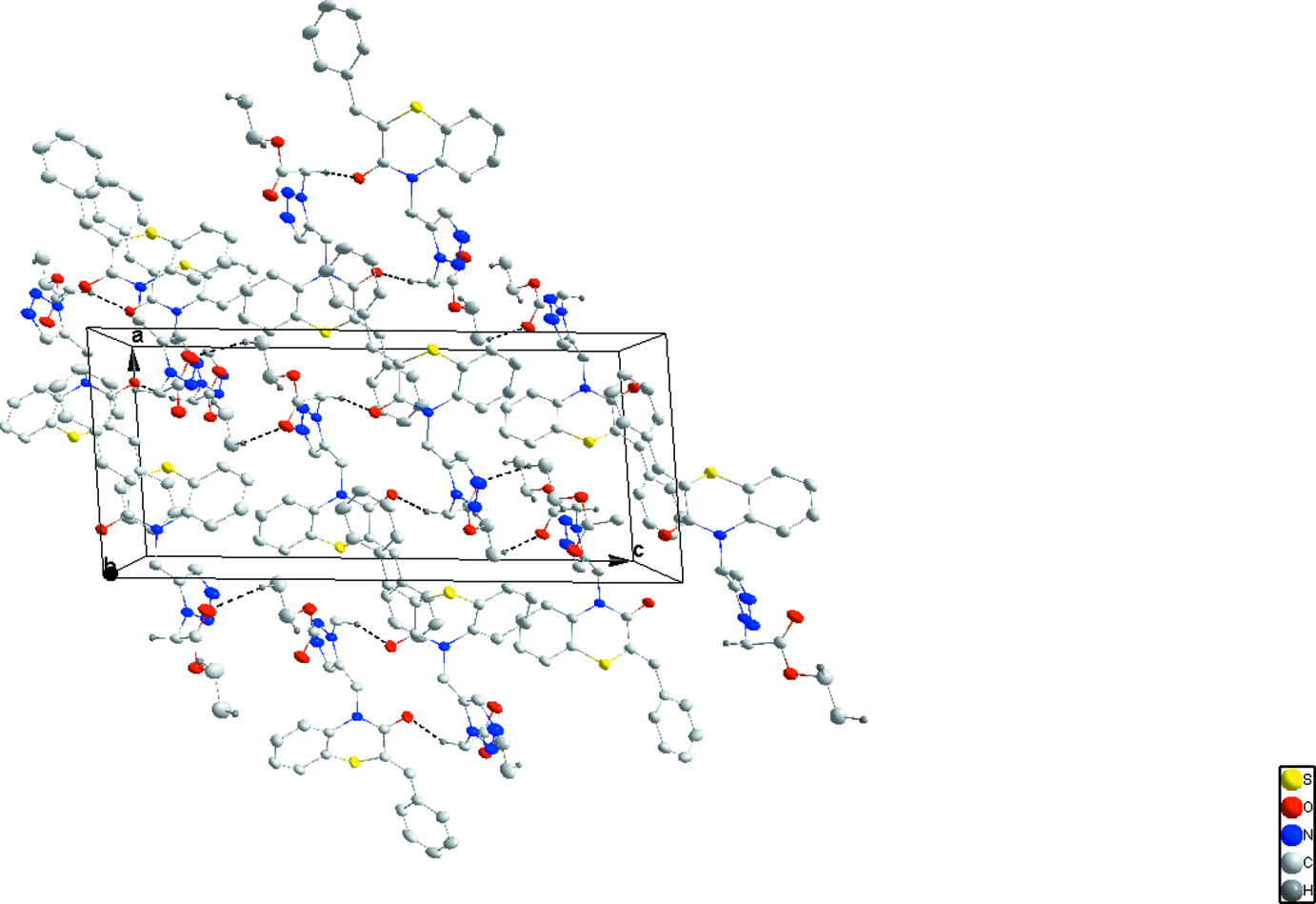

Supplement: Supplementary file 5 [file e-71-o1022-fig2.tif]
